# USEARCH 12: Open‐source software for sequencing analysis in bioinformatics and microbiome

**DOI:** 10.1002/imt2.236

**Published:** 2024-09-02

**Authors:** Yuanping Zhou, Yong‐Xin Liu, Xuemeng Li

**Affiliations:** ^1^ Zhanjiang Key Laboratory of Human Microecology and Clinical Translation Research, The Marine Biomedical Research Institute, College of Basic Medicine Guangdong Medical University Zhanjiang China; ^2^ Genome Analysis Laboratory of the Ministry of Agriculture and Rural Affairs, Agricultural Genomics Institute at Shenzhen Chinese Academy of Agricultural Sciences Shenzhen China

## Abstract

The well‐known bioinformatic software USEARCH v12 was open sourced. Its meaning encourages the microbiome research community to constantly develop excellent bioinformatic software based on the codes. The open source and popularization of artificial intelligence (AI) will make a better infrastructure for microbiome research.

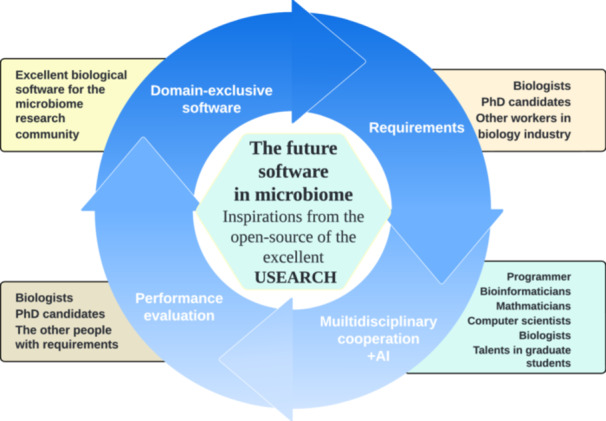

USEARCH is an excellent tool for biologists interested in sequence manipulation, microbial composition, and dynamics analysis in various niches, but the 64‐bit USEARCH has not been freely accessible for its commercial strategy in the past. Suddenly in 2024, the author of USEARCH open sourced the version 12 of USEARCH and provided all 64 bit USEARCH for free download, which indicated that the research community whoever conducts research in the field of microbiome could analyze their own data using all the excellent functions contained in USEARCH. To some extent, this can be amazing good news for the research community. However, with the open‐source version of USEARCH 12, the author only remained 22 critical functions that were previously contained in USEACH v11, which may encourage the community constantly to develop much more excellent software in the future. With the advent of artificial intelligence (AI) and multidisciplinary cooperation, especially cooperation between the talents from microbiology and from the mobile Internet industry, there will be more and more domain‐exclusive and excellent bioinformatic software for the microbiome research community.

## History of USEARCH in microbiome

**Figure 1:**
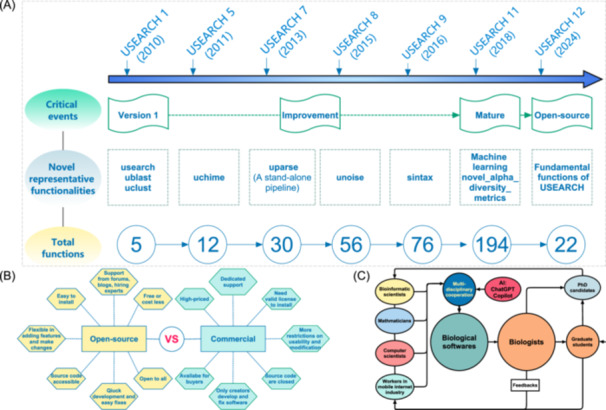
Past, current, and future of USEARCH. (A) The improvement history of different version of USEARCH and associated functionality components from the beginning to the open source. (B) Comparisons of characteristics between the open‐source and commercial software. (C) The development of pipeline or software in bioinformatic and microbiome in future.

In 2010, USEARCH v1 was published in *Bioinformatics* as an ultrafast sequence analysis tool, including usearch and uclust algorithms that are ~76× faster than megablast and ~27× faster than CD‐HIT, respectively [1]. In 2011, USEARCH v5 was released as the first stand‐alone program and included the cluster, query, makeudb, derep_fullseq, derep_subseq, uchime, and sort functions (Figure 1A). In 2018, USEARCH v11 was released, and 21 new commands (total commands 194) were compiled, such as functions for removal of primer sequence (fastx_trim_primer), random forest training and classifying (forest_train/forest_classify), and operational taxonomic unit (OTU) table manipulation (otutab_rare/otutab_samples/otutab_select, https://drive5.com/usearch/). Generally, the memory limitation of all the 32‐bit USEARCH is 4 GB, which should be extremely fit for running amplicon data under circumstances of dozens of samples. However, the mature USEARCH v11 may increase the limitation of data size. For example, many users when updating from v10 to v11 to rerun the project might report “‐‐‐Fatal error‐‐‐ File size too big for 32‐bit version,” even when manipulating file size only in hundreds of KB. This update leads many people to buy and use the 64 bit version. But more people will choose to replace it with other similar software, such as mothur [2], QIIME 2 [3], EasyAmplicon [4], or Parallel‐Meta Suite [5]. In 2024, USEARCH v12 is released and is the first open‐source version of USEARCH, which has removed many functions and kept only 22 fundamental commands, such as totally removing functions for OTU table manipulation or diversity analysis (Figure 1A). Meanwhile, the author of USEARCH released all 64‐bit versions of USEARCH on GitHub (https://github.com/rcedgar/usearch_old_binaries/) for free access. We also provide backup download links on GitHub (https://github.com/YongxinLiu/UsearchChineseManual). Thus, now users in the bioinformatics and microbiome research community could not only use the freely released 64 bit USEARCH (including USEARCH v11) to finish their own projects, but they could also integrate the remaining functions and learn from the open‐source codes of these functions in USEARCH v12 to create a brand new piece of software based on their own needs. It is noted that the maturation of USEARCH has gone through developing or upgrading a series of functionalities from USEARCH v1 to v11, to reach the state‐of‐the‐art software, some milestone functionality components for microbiome research such as uchime [6] for chimeric sequences identification in 2011 (cited 14,443 times, by August 1, 2024 and below is the same), and the stand‐alone pipeline uparse [7] for sequences quality filtering, trimming, and clustering in 2013 (cited 14,588 times), the unoise [8] for error filtering and correction of amplicon sequence variant in 2015 (cited 3800 times), and sintax [9] for sequences classification in 2016 (cited 725 times). Finally, the author of USEARCH nearly provided a complete resolution for the analysis of amplicon data in the microbiome research community by conquering real challenges.

## Commercial versus open source

As no fundings are supported from governments or any commercial organizations, the author of USEARCH as an independent investigator could only adopt the commercial strategy to get financial support from its users if they need to use the 64 bit version of USEARCH once upon a time. At the same time, the 32 bit version of USEARCH was still provided as a free nonprofit option. USEARCH has various advantages in comparison with its counterparts such as QIIME and Mothur, such as its less memory usage, easy installation, fast running speed, and accessible in different operating systems (Windows, Unix, MacOS). Therefore, it was regarded as one of the best software for amplicon analysis in the Microbiome. However, as the 64 bit USEARCH is commercial software ($1485 for commercial users and $885 for nonprofit users), it is a little expensive for a portion of researchers who only obtained limited funding. Therefore, an alternative software called VSEARCH [10] was published in 2016 and was cited 7910 times by 2024‐08‐01 due to the huge requirement from the research community for it replaced the main functions in USEARCH. The open source of USEARCH may ignite the passion of scientists in the microbiome research community to develop more excellent bioinformatic software soon. The advantages and disadvantages of commercial versus open source are show in Figure 1B.

## Tips for pipeline in bioinformatics and microbiome field

USEARCH is a very good choice for many users. First, the open‐source codes of USEARCH could be components for better software development in future. Second, the commands in all open released 64‐bit USEARCH could also be integrated into more pipelines to improve part of their low effective components in R/Python scripts. Third, the open source of USEARCH nourished the open‐source culture, which may improve the development environment for biologists to incorporate the capacity of AI [11] and cooperation among talents from different fields, such as biologist, PhD students, mathematicians, bioinformaticians, computer scientist, and talents, spilled over from the mobile Internet industry, to develop easier‐to‐use and efficient software in bioinformatics and microbiome in future (Figure 1C).

## AUTHOR CONTRIBUTIONS

**Yuanping Zhou**: Writing—original draft; writing—review and editing. **Yong‐Xin Liu**: Writing—review and editing; project administration; supervision; conceptualization; funding acquisition. **Xuemeng Li**: Funding acquisition; project administration; writing—review and editing.

## CONFLICT OF INTEREST STATEMENT

Yong‐Xin Liu holds the position of Executive Editor for iMeta.

## ETHICS STATEMENT

No animals or humans were involved in this study.

## Data Availability

All the impact and citations of USEARCH are statistics of Google Scholar by August 1, 2024. The open‐source USEARCH 12 is available on GitHub https://github.com/rcedgar/usearch12. The Chinese manual, amplicon pipeline and backup download links are available in GitHub https://github.com/YongxinLiu/UsearchChineseManual. Supporting Informations (graphical abstract, slides, videos, Chinese translated version, and update materials) may be found in the online DOI or iMeta Science http://www.imeta.science/.
